# Neonatal carrier: An easy to make alternative device to costly transport chambers

**DOI:** 10.4103/0971-9261.72436

**Published:** 2010

**Authors:** Milind Joshi, Sangram Singh, Anupama Negi, Tanmay Vyas, Vigya Chourishi, Anvesh Jain

**Affiliations:** Department of Pediatric Surgery, Sri Aurobindo Institute of Medical Sciences, Indore, Madhya Pradesh, India

**Keywords:** Cost, neonatal carrier, neonatal transport

## Abstract

The transport of sick neonates to the surgical centers or transportation within the center is an essential requirement of neonatal surgery. Neonatal transport incubators are costly, space occupying, and are not available at many places in the developing countries. We report here a cheap yet effective and easy to make, alternate neonatal carrier device.

## INTRODUCTION

The transportation of neonates has always been an important and crucial factor in the neonatal management. There has been an increasing tendency over the past two decades toward centralization of neonatal surgery and intensive care, not only for economic reasons, but also for availability of specialized personnel. The transport of sick infants to the surgical centers is, therefore, necessary.[1–3]

In India, where the facilities of surgical care are not commonly available, it is not feasible to expect good neonatal transport facilities. Hence, the need for a cheap yet effective neonatal carrier is always felt as the presently available transporters are costly, space occupying, and are not an easy option in our area. We have developed an easy to make, cheap but effective neonatal carrier for transporting the neonates from and to the neonatal care unit and operating room.

## HOW TO MAKE IT?

**Figure 1:**
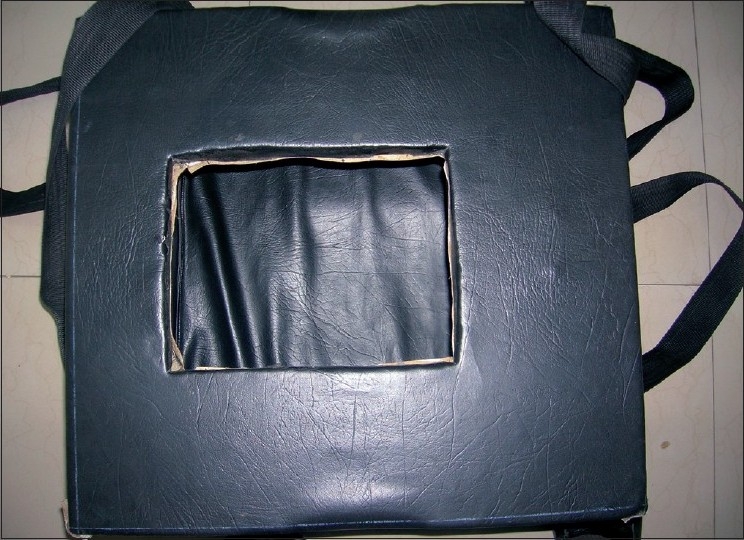
Neonatal carrier with the cover

**Figure 2:**
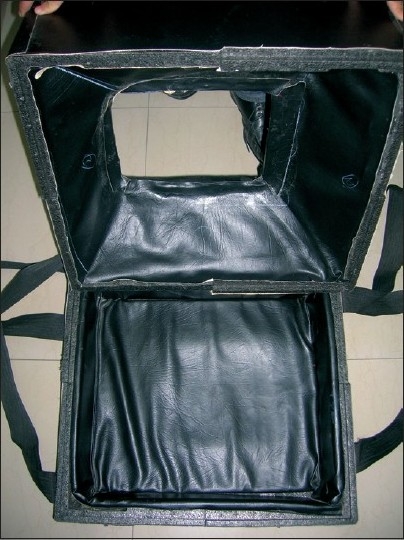
Neonatal carrier with inner surface

**Figure 3:**
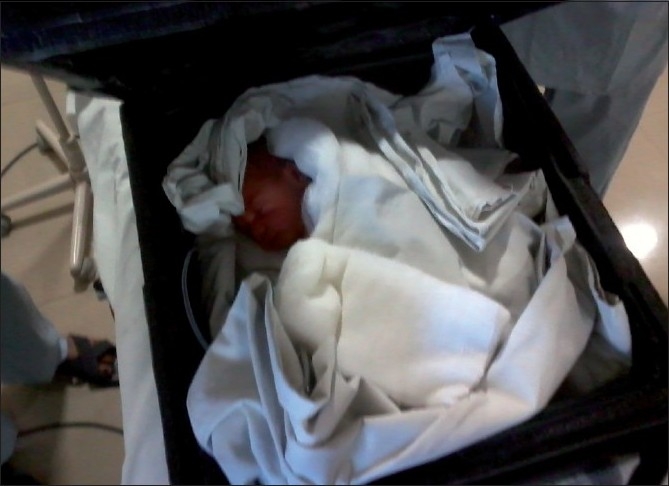
Neonatal carrier with a neonate

This neonatal carrier is very easy to make at any health center. It consists of cardboards of approximately 40 × 40 cm^2^. It is cushioned with thermacoal from inside which acts as a shock absorber and also as an insulator. The inner surface is lined by black color paper [Figure 1]. The cover is also made of thermacol of the same size which has an opening of about 15 × 15 cm^2^ in the center so that the intravenous fluid tubing and oxygen tubings can be brought out [Figure 2]. The carrier has two slings attached to it, one on each side to carry it by hands [Figure 3]. It can also be kept on a patient shifting trolley as it is very stable on the base. The approximate weight of the carrier is 300 g, and it costs about Rs. 100.

## DISCUSSION

This neonatal carrier has the following advantages: (1) It is easy to make. (2) It is cheap and effective. (3) It can be used for transportation of very low birth weight neonates also. (4) It is very handy and can be carried by single person by hand or on patient shifting trolley. The carrier is being used to transport the neonates to operation room and back to neonatal care ward without any problems about hypothermia. We are able to transport even the very low birth weight babies with this carrier. There was no temperature difference between pre- and post transportation. Probably, this was due to the short distance between our neonatal intensive care and operation room which is about 100 m. However, even when we transported babies from the pediatric medicine department in this box, the temperature difference was not more than 0.5° C as compared to the transportation in the warm wraps where the difference was 1° C on an average and was significant. We have used this box for neonatal transport in about 60 neonates till now.

The hypothesis of thermo equilibrium simply follows the Newton’s law of cooling which states that the rate of cooling is directly proportional to the fourth power of temperature difference between the two objects. Our neonatal carrier simply delays diffusion of the heat to the external environment as it is open only from the top and covered with black color paper which has natural tendency to retain the heat. As compared to only covering with warm wraps, this carrier box slows the rate of cooling of the neonate.

This neonatal transport carrier cannot replace the modern state of the art machinery, but it can be an alternative to the presently used carriers such as wooden box or cardboard box which is commonly used for the transport of the neonates from the periphery. This carrier can be very easily made at the primary health centers and rural areas at cheaper cost. We feel that this carrier can be successfully utilized by the peripheral centers for the transportation of a short duration, where the facility of thermostatic transporters may not be available.
